# The tumor suppressor p53 is a negative regulator of the carcinoma-associated transcription factor FOXQ1

**DOI:** 10.1016/j.jbc.2024.107126

**Published:** 2024-03-01

**Authors:** Giulia Pizzolato, Lavanya Moparthi, Pierfrancesco Pagella, Claudio Cantù, Pádraig D’Arcy, Stefan Koch

**Affiliations:** 1 Wallenberg Centre for Molecular Medicine (WCMM), Linköping University, Linköping, Sweden; 2 Department of Biomedical and Clinical Sciences (BKV), Linköping University, Linköping, Sweden

**Keywords:** FOXQ1, p53, proteomics, GloPro, gene transcription, forkhead box, colorectal cancer, gene regulation

## Abstract

The forkhead box family transcription factor FOXQ1 is highly induced in several types of carcinomas, where it promotes epithelial-to-mesenchymal transition and tumor metastasis. The molecular mechanisms that lead to *FOXQ1* deregulation in cancer are incompletely understood. Here, we used CRISPR–Cas9-based genomic locus proteomics and promoter reporter constructs to discover transcriptional regulators of *FOXQ1* and identified the tumor suppressor p53 as a negative regulator of *FOXQ1* expression. Chromatin immunoprecipitation followed by quantitative PCR as well as complementary gain and loss-of-function assays in model cell lines indicated that p53 binds close to the transcription start site of the FOXQ1 promoter, and that it suppresses *FOXQ1* expression in various cell types. Consistently, pharmacological activation of p53 using nutlin-3 or doxorubicin reduced FOXQ1 mRNA and protein levels in cancer cell lines harboring wildtype p53. Finally, we observed that p53 mutations are associated with increased *FOXQ1* expression in human cancers. Altogether, these results suggest that loss of p53 function—a hallmark feature of many types of cancer—derepresses *FOXQ1*, which in turn promotes tumor progression.

The forkhead box (FOX) family transcription factor FOXQ1 is a putative carcinoma oncogene that is highly induced in several types of cancer. FOXQ1 has been shown to promote epithelial-to-mesenchymal transition (EMT) and metastasis and to increase the resistance of cancer cells to chemotherapeutic drugs (1, 2, 3). Accordingly, high *FOXQ1* levels are an independent prognostic factor for worse overall survival in, for example, colorectal, lung, stomach, and liver cancer (4, 5, 6, 7). It is therefore of considerable interest to delineate the molecular mechanisms that regulate *FOXQ1* expression during carcinogenesis. Previous studies suggest that *FOXQ1* induction may be driven by various mitogenic signaling pathways, including Wnt/β-catenin, YAP/TAZ, and FGFR1/ERK2 signaling (8, 9, 10); however, it is unlikely that these observations can sufficiently account for the increase in *FOXQ1* levels across different cancer types.

We therefore used an unbiased CRISPR–Cas9-based proximity proteomics approach to screen for new transcriptional regulators of FOXQ1 and identified the p53 protein as a candidate inhibitor of *FOXQ1* expression. p53 is a prominent tumor suppressor that responds to multiple cellular stresses to regulate the transcription of target genes involved in cell cycle arrest and apoptosis (11). Loss-of-function mutations in the *TP53* gene, which encodes p53, are found in more than half of all human cancers (11). Loss of p53 activity causes aberrant cell proliferation and apoptosis resistance through the transcriptional rewiring of affected cells (12). Here, we show that p53 engages the FOXQ1 promoter and suppresses its activity, and that common cancer-associated *TP53* mutations result in the derepression of *FOXQ1*. These observations suggest that the increased *FOXQ1* expression observed in many cancers may be explained in part by loss of function of the p53 tumor suppressor, which may have important implications for tumor progression and metastasis.

## Results

### Genomic locus proteomics identifies new transcriptional regulators of FOXQ1 expression

**Figure 1:**
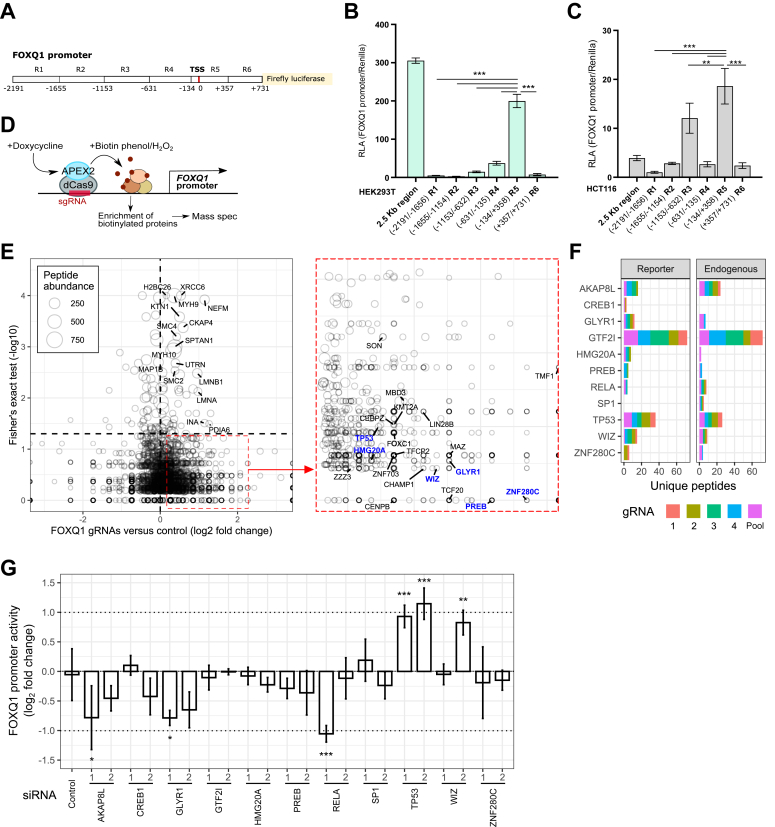
**Proximity proteomics highlight potential *FOXQ1* regulators.***A*, schematic representation of reporter plasmids covering fragments of the human *FOXQ1* promoter (R1 through R6). Distances from the transcription start site (TSS) are indicated. In addition, a full-length reporter construct (2.5 kb region) was used. *B* and *C*, luciferase assays in 293T (*B*) and HCT116 cells (*C*) using the FOXQ1 promoter reporter plasmids. Graphs show mean and SD of normalized luciferase values of n = 3 independent experiments (ANOVA with Tukey’s post hoc test, ∗∗*p* < 0.01, ∗∗∗*p* < 0.001). *D*, schematic workflow of the Genomic Locus Proteomics (GLoPro) assay. Doxycycline-induced Caspex (dCas9-APEX2) protein was guided to the *FOXQ1* promoter by independent gRNAs. Biotinylation of proteins was triggered by the addition of hydrogen peroxide, and enrichment of these proteins was performed by streptavidin pulldown. Proteins were then identified by mass spectrometry. *E*, volcano plot of protein hits identified by GLoPro in the 293T Caspex stable cell line following transfection of the full-length FOXQ1 promoter reporter construct across the four guide RNAs compared with the no-guide RNA control. On the *right*, transcription factors enriched at the FOXQ1 promoter are highlighted. Proteins further investigated in subsequent experiments are indicated in *blue*. *F*, shortlist of potential transcriptional regulators of *FOXQ1* expression, as identified by GLoPro. Unique peptides of proteins enriched at the promoter reporter (*left graph*) and the endogenous *FOXQ1* promoter (*right graph*, Fig. S1*F*) are indicated. *G*, FOXQ1 promoter activity assay in HCT116 cells transfected with the indicated siRNAs. The graph shows mean and SD of normalized luciferase values of n = 3 independent experiments (ANOVA with Dunnett's post hoc test, ∗*p* < 0.05, ∗∗*p* < 0.01, ∗∗∗*p* < 0.001). *Dashed lines* indicate a twofold change in activity.

*FOXQ1* levels are dramatically increased in various types of cancer, including colorectal cancer (CRC). To gain further insight into the regulation of *FOXQ1* expression, we initially used a FOXQ1 promoter reporter plasmid (13), which encompasses a ∼2.5 kb segment upstream of the transcription start site (TSS) of the human FOXQ1 promoter (Fig. 1*A*). In addition, we generated luciferase reporter plasmids containing consecutive ∼500 bp fragments of the FOXQ1 promoter (−2191 to +731) (Fig. 1*A*). Reporter assays in noncancer 293T and multiple CRC cell lines identified a highly active promoter region close to the TSS (R5, −134 to +357) in all tested cells (Figs. 1, *B* and *C*, and S1, *A* and *B*), whereas other promoter regions were considerably less active.

To get an unbiased overview of transcriptional regulators associated with the R5 region of the FOXQ1 promoter, we used CRISPR–Cas9-based genomic locus proteomics (GLoPro (14)) in stably transfected 293T cells expressing a doxycycline-inducible dCas9-APEX2 protein (Figs. 1*D* and S1, *C*–*E*). We transfected these cells with four validated nonoverlapping guide RNAs targeting the FOXQ1 promoter close to the TSS (13) and performed proximity biotinylation following doxycycline treatment. For validation and to account for potential epigenetic modification of the FOXQ1 promoter (2), these assays were performed on the endogenous FOXQ1 promoter, that is, untransfected cells, as well as in cells transfected with the full-length promoter construct described previously (Figs. 1*E* and S1*F* and supporting datasets 1 and 2). Likely because of the intrinsically low sensitivity and specificity of the GLoPro method (14), proteins that were significantly enriched compared with no guide control mainly encompassed high abundance proteins such as lamins and histones (Figs. 1*E* and S1*F*). However, inspection of the proteomics data revealed numerous transcription factors that were (nonsignificantly) enriched at the FOXQ1 promoter and that may thus be *FOXQ1* regulators (Fig. 1*F*). We selected several of these candidate regulators based on their relevance for cancer biology and tested their effect by loss-of-function assays in HCT116 CRC cells (Fig. 1*G*). Depletion of *TP53* with two independent siRNAs significantly increased FOXQ1 promoter activity, whereas *GLYR1* depletion reduced promoter activity. *RELA*, *WIZ*, and *AKAP8L* depletion altered FOXQ1 promoter activity as well, but this effect was only observed with one siRNA. We conclude that GLoPro can be used to prioritize candidate regulators of *FOXQ1* expression.

### p53 engages the FOXQ1 promoter and represses *FOXQ1* expression

**Figure 2:**
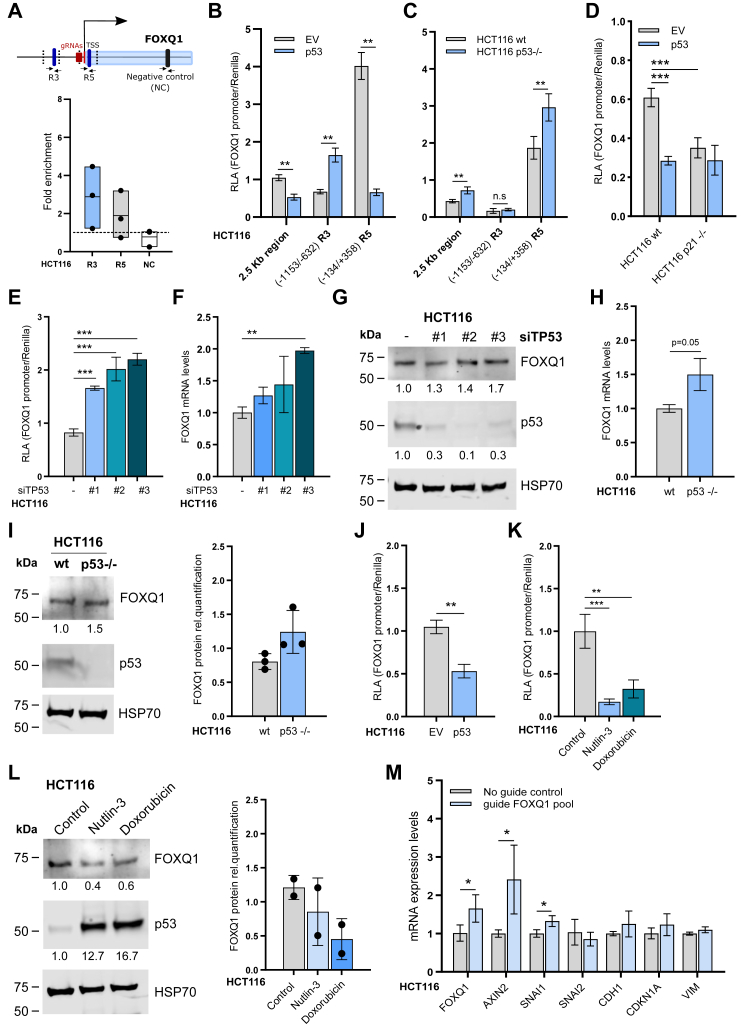
**p53 is a transcriptional repressor of *FOXQ1*.***A*, ChIP–qPCR assay of endogenous p53 in HCT116 cells. Primers used in this assay are illustrated in the schematic above and targeted the regions R3 and R5 of the FOXQ1 promoter region. Results are displayed as fold enrichment compared with the control antibody across three independent experiments. *B*, luciferase assay in HCT116 cells upon p53 overexpression using the indicated FOXQ1 promoter reporter constructs. *C*, luciferase assay in wildtype or p53 KO (−/−) HCT116 cells. *D*, luciferase assay in HCT116 p21 KO (−/−) cells using the FOXQ1 promoter construct. Graphs show mean and SD of normalized luciferase values of n = 3 independent experiments. (ANOVA with Tukey's post hoc test, ∗∗∗*p* < 0.001). *E*, luciferase assay in HCT116 cells using the full-length FOXQ1 reporter plasmid upon silencing of *TP53* with three independent siRNAs. Graphs show mean and SD of normalized luciferase values of n = 3 independent experiments. (ANOVA with Dunnett's post hoc test, ∗∗*p* < 0.01, ∗∗∗*p* < 0.001). *F*, qPCR analysis of *FOXQ1* mRNA levels in HCT116 cells upon silencing of *TP53*. *FOXQ1* expression was normalized to *HPRT1* housekeeping gene. Data are displayed as mean and SD from experiments with biological triplicates and technical duplicates (ANOVA with Dunnett's post hoc test, ∗∗*p* < 0.01). *G*, immunoblot analysis of FOXQ1 protein levels in HCT116 upon silencing of *TP53*. FOXQ1 and p53 levels were normalized to HSP70 loading control, and the relative protein levels in this and the following panels are indicated below the blots. *H*, qPCR analysis of *FOXQ1* mRNA levels in HCT116 wildtype and p53 KO cell lines. FOXQ1 gene expression was normalized to *GAPDH* housekeeping gene. Data are displayed as mean and SD from biological triplicates and technical duplicates (Welch’s *t* test). *I*, immunoblot analysis of FOXQ1 protein levels in HCT116 wildtype and p53 KO cell lines. Relative abundance of FOXQ1 protein from n = 3 independent experiment is reported. *J*, luciferase assay in HCT116 cells using the full-length FOXQ1 reporter plasmid after overexpression of p53 wildtype plasmid. Mean and SD of normalized luciferase values of n = 2 independent experiments. (Welch’s *t* test, ∗∗*p* < 0.01). *K*, luciferase assay in HCT116 cells using the FOXQ1 reporter plasmid after treatment with 10 μM Nutlin-3 or 1 μM doxorubicin for 24 h. Mean and SD of normalized luciferase values of n = 3 independent experiments (ANOVA with Dunnett's post hoc test, ∗∗*p* < 0.01, ∗∗∗*p* < 0.001). *L*, immunoblot analysis of FOXQ1 protein levels after treatment with 10 μM Nutlin-3 or 1 μM doxorubicin for 24 h. Relative abundance of FOXQ1 protein from n = 2 independent experiment is reported. *M*, CRISPR activation of *FOXQ1* in HCT116 cells followed by qPCR of selected FOXQ1 target genes. Gene expression was normalized to *GAPDH* housekeeping control. Data are displayed as mean and SD from experiments with biological triplicates and technical duplicates (Welch’s *t* test, ∗*p* < 0.05). ChIP, chromatin immunoprecipitation; qPCR, quantitative PCR.

We focused our subsequent efforts on *TP53*, since loss of p53 activity is a hallmark feature of many types of cancer. To validate the GLoPro data, we first performed chromatin immunoprecipitation followed by quantitative PCR (ChIP–qPCR) in HCT116 cells, which harbor wildtype p53 (Fig. 2*A*). For this, we designed primer pairs within the labeling radius of dCas9-APEX2 of approximately 400 bp (14) as well as negative control primers in the FOXQ1 gene body. Although there was some variability between individual experiments, we observed p53 enrichment in both the R3 and R5 regions compared with control (Fig. 2*A*). Consistently, analysis of public ChIP-Seq data curated in the ReMap 2022 database (15) supported binding of p53 to the FOXQ1 promoter across multiple cell types, including SW480 CRC cells (Fig. S2*A*). Although sequence inspection of these regions revealed no consensus p53-binding motifs, it is known that numerous genes contain low-affinity p53 response elements close to their TSS (16). We therefore mutated two putative p53-binding sites in the R5 region of the 2.5 kb promoter reporter plasmid, which did not affect the inhibitory action of p53 but rather reduced basal reporter activity by ∼25% (Fig. S2, *A* and *B*). We then performed promoter reporter assays in HCT116 using the FOXQ1 promoter fragments described previously. *TP53* gain-of-function and loss-of-function experiments confirmed that p53 mainly inhibits the R5 region of the promoter, although we also observed activation of the R3 fragment in the presence of p53 (Figs. 2, *B* and *C*, and S2*C*). Moreover, we assessed FOXQ1 promoter activity in HCT116 cells lacking *CDKN1A* (p21), a target gene of p53 that was previously shown to mediate the repression of various genes (17), including the related transcription factor FOXM1 (18). FOXQ1 promoter reporter activity following p53 overexpression was comparable between p21-deficient and control cells (Fig. 2*D*). However, loss of p21 by itself resulted in a strong reduction of FOXQ1 promoter activity, suggesting that p21 may be a positive regulator of *FOXQ1* expression in HCT116 cells.

Next, we investigated how p53 affects *FOXQ1* expression. *TP53* depletion using three independent siRNAs increased FOXQ1 promoter reporter activity, gene expression, and protein levels in HCT116 cells, consistent with our earlier findings (Fig. 2, *E*–*G*). Similar results were obtained with SW48 CRC cells that also harbor normal p53, although one siRNA appeared to be ineffective in these cells (Fig. S2, *D* and *E*). Moreover, FOXQ1 mRNA and protein levels were also increased in HCT116 *TP53* knockout cells compared with their wildtype counterpart (Fig. 2, *H* and *I*). Conversely, p53 overexpression reduced FOXQ1 promoter activity in HCT116 and SW48 cells (Figs. 2*J*, and S2*F*). Finally, we used nutlin-3, an inhibitor of the E3 ubiquitin ligase MDM2, as well as the chemotherapeutic agent doxorubicin to stabilize and activate p53 (19, 20). In agreement with our previous results, both drugs decreased FOXQ1 promoter activity and protein levels in HCT116 and SW48 cells (Figs. 2, *K* and *L*, and S2, *G* and *H*). We in addition repeated these experiments in HeLa (cervical cancer) cells with wildtype p53 and obtained consistent results (Fig. S2, *I*–*M*); p53 depletion resulted in significantly increased FOXQ1 promoter activity, mRNA, and protein levels, whereas p53 overexpression caused repression of FOXQ1 promoter activity and mRNA levels. Finally, we repeated these experiments in 293T cells, in which p53 is stabilized but inactivated by the SV40 large T antigen (21, 22). In these cells, p53 depletion marginally increased FOXQ1 promoter reporter activity but did not increase FOXQ1 protein levels (Fig. S2, *N* and *O*). Conversely, ectopic overexpression of p53 substantially reduced FOXQ1 promoter activity (Fig. S2*P*). Moreover, doxorubicin, but not nutlin-3, reduced FOXQ1 promoter activity and protein levels in 293T cells (Fig. S2, *Q* and *R*), which may be explained by their differential effect on p53 stabilization in these cells.

Across all experiments, *FOXQ1* induction following p53 loss of function was low, averaging approximately 50%. Therefore, we performed CRISPR activation assays in HCT116 cells to investigate whether modest changes in *FOXQ1* expression would affect the transcription of FOXQ1 target genes implicated in tumorigenesis and EMT. Indeed, FOXQ1 induction to the extent observed following p53 loss of function was sufficient to significantly increase the expression of *AXIN2*, a Wnt/β-catenin pathway inhibitor, and the EMT-related transcription factor *SNAI1* (Fig. 2*M*). We thus conclude that p53 is a biologically relevant repressor of FOXQ1-dependent gene transcription in multiple cell types.

### Mutant p53 is associated with increased FOXQ1 expression in cancers

**Figure 3:**
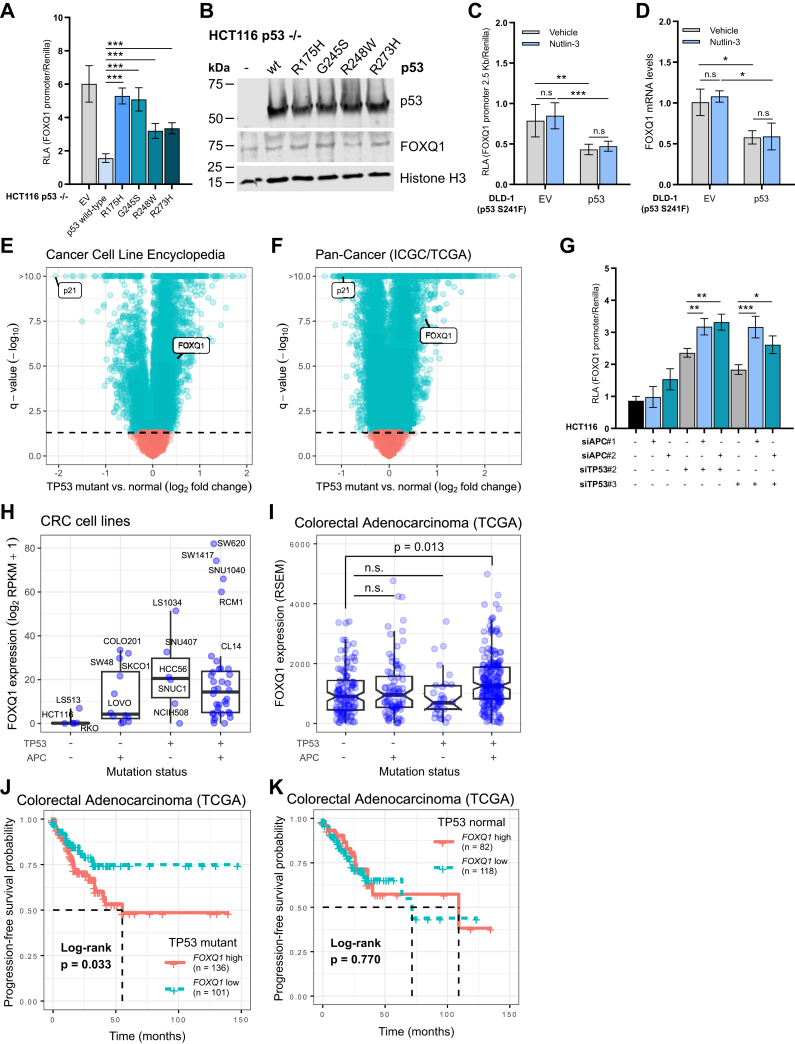
**Mutational inactivation of p53 is associated with increased FOXQ1 expression in cancers.***A*, luciferase assay in HCT116 p53 KO cells upon overexpression of wildtype p53 and four common p53 mutants (missense mutations are indicated on the *x*-axis) using the FOXQ1 promoter reporter construct. Mean and SD of normalized luciferase values of n = 2 independent experiments (ANOVA with Tukey’s post hoc test, ∗∗∗*p* < 0.001). *B*, immunoblot analysis of p53 wildtype and p53 mutants and FOXQ1 protein levels in HCT116 p53 KO cells. FOXQ1 and p53 levels were normalized to histone H3 loading control. *C*, luciferase assay in DLD-1 cells harboring mutant p53 (S241F) using the FOXQ1 reporter plasmid after transient transfection with empty vector (EV) or wildtype p53. Where indicated, cells were treated with 10 μM Nutlin-3 for 24 h. Mean and SD of normalized luciferase values of n = 2 independent experiments (ANOVA with Tukey’s post hoc test, ∗∗*p* < 0.01, ∗∗∗*p* < 0.001 or n.s. = not significant). *D*, qPCR analysis of *FOXQ1* mRNA levels in DLD-1 cells after treatment with 10 μM Nutlin-3 for 24 h and overexpression of wildtype p53. FOXQ1 gene expression was normalized to *GAPDH* housekeeping gene (ANOVA with Tukey's post hoc test, ∗*p* < 0.05 or n.s = not significant). *E*, gene expression analysis in the Cancer Cell Line Encyclopedia. Across all cell lines, *TP53* mutation was associated with increased *FOXQ1* expression. Decreased expression of the p53 target p21 highlights *TP53* loss of function. The *dashed line* indicates a *q* value threshold of 0.05. *F*, gene expression analysis in a Pan-Cancer dataset. *TP53* mutation was associated with higher *FOXQ1* levels in cancer patients. *G*, luciferase assay in HCT116 cells using the FOXQ1 reporter plasmid upon silencing of *APC* and/or *TP53* by two independent siRNAs. Mean and SD of normalized luciferase values of n = 2 independent experiments (ANOVA with Tukey's post hoc test, ∗∗*p* < 0.01, ∗∗∗*p* < 0.001). *H*, *FOXQ1* expression analysis in colorectal cancer (CRC) cell lines included in the Cancer Cell Line Encyclopedia. *TP53* mutation was associated with higher *FOXQ1* levels irrespective of concomitant *APC* mutations. Representative cell lines in each group are highlighted. *I*, *FOXQ1* expression analysis in Colorectal Adenocarcinoma. Concomitant *TP53* and *APC* mutation was associated with increased *FOXQ1* levels. *J* and *K*, progression-free survival in patients with CRC stratified by *FOXQ1* expression. Elevated FOXQ1 levels were associated with worse prognosis in *TP53* mutant (*J*) but not *TP53* normal cases (*K*). qPCR, quantitative PCR.

Defects in the p53 pathway are present in the majority of cancers (11). To follow up on the preceding results, we tested the effect of four of the most common p53 missense mutants (R175H, G245S, R248W, R273H, all located in the DNA-binding domain) (23) expressed in HCT116 p53 knockout cells. Compared with wildtype p53, all tested p53 mutants were significantly less effective at repressing FOXQ1 promoter activity (Fig. 3, *A* and *B*). We then tested DLD-1 CRC cells that harbor another clinically relevant inactivating missense mutation of p53 (S241F). In this experiment, we investigated the changes in FOXQ1 expression upon treatment with nutlin-3, rescue of wildtype p53 expression, or a combination of both. As expected, nutlin-3 treatment did not change FOXQ1 promoter activity and mRNA levels in these cells, whereas overexpression of wildtype p53 reduced both (Fig. 3, *C* and *D*). We then analyzed public datasets of cancer cell lines and human cancer samples. Both in the Cancer Cell Line Encyclopedia (24) and the Pan-Cancer Analysis of Whole Genomes project (25, 26), which comprise thousands of samples across numerous tissues of origin, mutation of *TP53* was associated with significantly higher FOXQ1 mRNA levels (Fig. 3, *E* and *F*).

Our results so far indicated that p53 does not repress FOXQ1 actively, but that it acts as a roadblock to *FOXQ1* induction by other, activating transcription factors. In CRC, sequential loss of function of *APC* (a core inhibitor of Wnt/β-catenin signaling) and *TP53* is common during adenoma-to-carcinoma progression (27), and β-catenin signaling has been shown to induce *FOXQ1* expression in CRC cells (8) (Fig. S3, *A* and *B*). We, therefore, investigated if these combined genetic lesions would result in higher *FOXQ1* levels. Indeed, concomitant depletion of *TP53* and *APC* in HCT116 cells was associated with significantly higher *FOXQ1* promoter activity compared with knockdown of either gene alone (Fig. 3*G*). Among CRC cell lines included in the Cancer Cell Line Encyclopedia, increased *FOXQ1* levels were observed in cells with individual or combined mutation of *TP53* and *APC*, although these changes did not reach statistical significance (Fig. 3*H*). Moreover, in human CRC samples, concomitant mutation of *TP53* and *APC* was associated with significantly increased FOXQ1 mRNA levels (Fig. 3*I*). Finally, we investigated if *FOXQ1* induction following p53 loss of function may impact clinical outcomes in this CRC dataset. Neither increased *FOXQ1* expression nor *TP53* mutation alone had a significant effect on overall or progression-free survival (all log-rank *p* values >0.05). In contrast, elevated *FOXQ1* levels were associated with worse progression-free survival only in a *TP53* mutant background (Fig. 3, *J* and *K*). Although these observations should be interpreted with caution as they do not account for confounding factors and do not establish causality, they suggest that FOXQ1 contributes to CRC progression following p53 inactivation.

## Discussion

In summary, we used unbiased GLoPro to identify the tumor suppressor p53 as a negative regulator of the carcinoma-associated transcription factor FOXQ1. The mechanism by which p53 controls FOXQ1 appears to be complex and requires further investigation. On the one hand, both our own experiments and previous ChIP-Seq studies (15) support that p53 engages the FOXQ1 promoter close to its TSS. On the other hand, we could not identify p53-binding sites in the FOXQ1 promoter that may facilitate direct gene regulation, although low-affinity binding sites may be obfuscated by motif degeneration (16, 28). p53 is thought to act mainly as a transcriptional activator, but different modes of p53-dependent gene repression have been described, such as steric interference, inactivation of transcription cofactors, and the recruitment of histone deacetylases (16). In addition, p53 represses numerous other genes indirectly *via* its target gene p21 (17), a prominent transcriptional regulator that has also been shown to inhibit the related protein FOXM1 (18). Unexpectedly, loss of p21 reduced FOXQ1 promoter reporter activity in HCT116 cells, although it remains to be determined whether this observation applies to the endogenous promoter as well. Moreover, we found that p53 increases the activity of some parts of the FOXQ1 promoter, consistent with its role as a transcription activator. Thus, it is possible that p53 has both positive (*e.g.*, by direct binding of the R3 region) and negative effects on FOXQ1 promoter activity (*e.g.*, by indirect repression of the R5 region *via* p21), which in sum inhibit *FOXQ1* gene transcription because of the different basal activities of the individual regions.

The expression of *FOXQ1* is controlled by a collective of transcription factors, whose stoichiometry determines the activity of the FOXQ1 promoter in different tissues (3, 8, 9, 10). Accumulating mutations in different oncogenic pathways may thus have synergistic effects on *FOXQ1* expression, with p53 potentially acting as a common repressor that keeps *FOXQ1* levels low in normal tissues. FOXQ1 promotes EMT and metastasis in various carcinomas and may be a therapeutic target in, for example, breast and CRC (29, 30). On the other hand, FOXQ1 may also act as a tumor suppressor in other types of cancer such as melanomas, which is thought to depend on the recruitment of different transcription cofactors (31). Even though the effect of p53 on *FOXQ1* levels is modest, we find that small increases in *FOXQ1*, similar to those observed following p53 loss of function, are sufficient to alter the expression of FOXQ1 target genes implicated in tumor progression and EMT (32, 33). Thus, even minor changes in *FOXQ1* levels in cancer cells with p53 mutations may contribute to a shift toward a more aggressive phenotype and ultimately result in a worse prognosis.

## Experimental procedures

Detailed experimental procedures, including protocols for GLoPro, ChIP–qPCR, CRISPR activation, immunoblotting, and *in silico* analyses, can be found in the supporting information.

### Cell lines

293T, HCT116, HCT116 p53−/−, HCT116 p21−/−, SW48, and HeLa cells were cultured in Dulbecco's modified Eagle's medium supplemented with 10% fetal bovine serum, 2 mM glutamine, and 1% penicillin and streptomycin at 37 °C/5% CO_2_. DLD-1 cells were cultured in McCoy’s 5A media supplemented with 10% fetal bovine serum and 1% penicillin–streptomycin. 293T-Caspex stable cells were generated by transfecting a doxycycline-inducible Caspex plasmid (see later). The absence of mycoplasma infection was confirmed by analytical qPCR (Eurofins Genomics).

### Antibodies and reagents

The following antibodies were used: mouse anti-p53 (DO-1, sc-126) from Santa Cruz Biotechnology; rabbit anti-FOXQ1 (PA5-40772) and rabbit anti-Histone H3 (PA5-16183) from Invitrogen; mouse anti-FLAG M2 (F3165) from Sigma–Aldrich; rabbit anti-HSP70 (AF1663) from R&D Systems; and IRDye 800CW Streptavidin from LI-COR. Nutlin-3 and doxorubicin were purchased from Sigma–Aldrich. siRNAs were obtained from Integrated DNA Technologies.

### Plasmids and molecular cloning of FOXQ1 reporters

Plasmids included in this study are p53 wildtype (34), Caspex plasmid (deposited by Steven Carr & Samuel Myers (14); Addgene plasmid #97421), and dCas9-VP64-p65-Rta (deposited by George Church (35); Addgene plasmid #63798). The 2.5 kb FOXQ1 promoter reporter plasmid has been previously described (13). The complete region of FOXQ1 promoter was divided into shorter fragments (see supporting information). Missense mutations in the p53 wildtype plasmid and the FOXQ1 promoter reporter were generated by PCR-based mutagenesis (36). All plasmids were validated by partial sequencing (Eurofins Genomics).

### Public data analysis

The following public datasets were used for analysis: Cancer Cell Line Encyclopedia (Broad Institute (24)), Pan-Cancer analysis of whole genomes (ICGC/TCGA (25, 26)), and Colorectal Adenocarcinoma (TCGA (25, 26)).

### Statistical analysis

Data are shown as mean with standard deviation. Each experiment included controls (*e.g.*, empty backbone plasmid, scrambled siRNA, and substance carriers) at identical concentrations. Statistical tests are indicated in the figure legends and were carried out in R, version 4.2.1.

## Data availability

The mass spectrometry proteomics data have been deposited to the ProteomeXchange Consortium *via* the PRIDE (37) partner repository with the dataset identifier PXD047868 and 10.6019/PXD047868.

## Supporting information

This article contains supporting information (13, 14, 15, 24, 25, 26, 34, 35, 36, 37, 38, 39, 40).

## Conflict of interest

The authors declare that they have no conflicts of interest with the contents of this article.
