# Supplementary material for: The tumor suppressor p53 is a negative regulator of the carcinoma-associated transcription factor FOXQ1
Source: J Biol Chem. 2024 Mar 1;300(4):107126. doi: 10.1016/j.jbc.2024.107126 (PMC10981115; doi:10.1016/j.jbc.2024.107126)
Supplement: Supporting Information [file mmc1.pdf]

Supporting Information

The tumour suppressor p53 is a negative regulator of the carcinoma-associated transcription factor FOXQ1

Giulia Pizzolato, Lavanya Moparthi, Pierfrancesco Pagella, Claudio Cantù, Pádraig D'Arcy, and Stefan Koch

Materials included:

Supporting experimental procedures

Supplemental figure S1: Additional controls for FOXQ1 promoter reporter and GLoPro experiments.

Supplemental figure S2: p53 is a negative regulator of *FOXQ1* expression.

Supplemental figure S3: FOXQ1 is a target of the Wnt/β-catenin pathway.

SUPPORTING EXPERIMENTAL PROCEDURES

Cell lines

293T, HCT116, HCT116 p53-/-, HCT116 p21-/-, SW48, and HeLa cells were cultured in DMEM supplemented with 10% fetal bovine serum (FBS), 2 mM glutamine, and 1% penicillin and streptomycin at 37°C / 5% CO2. DLD-1 cells were cultured in McCoy's 5A media supplemented with 10% FBS and 1% (v/v) penicillin/streptomycin. 293T Caspex stable cells were generated by transfection of a doxycycline-inducible Caspex expression vector (see "Plasmids and molecular cloning of FOXQ1 reporters", below) followed by puromycin selection and generation of clonal lines. Absence of mycoplasma infection was confirmed by analytical qPCR (Eurofins Genomics, Ebersberg, Germany).

Antibodies and reagents

The following antibodies were used: mouse anti-p53 (DO-1, sc-126) from Santa Cruz Biotechnology (Dallas, TX, USA); rabbit anti-FOXQ1 (PA5-40772) and rabbit anti-Histone H3 (PA5-16183) from Invitrogen (Waltham, MA, USA); mouse anti-Flag M2 (F3165) from Sigma Aldrich (St Louis, USA); rabbit anti-HSP70 (AF1663) from R&D Systems; IRDye 800CW Streptavidin was from LI-COR Biosciences (Lincoln, NE, USA). Nutlin-3 and doxorubicin were purchased from Sigma Aldrich.

Plasmids and molecular cloning of FOXQ1 reporters

Plasmids included in this study are: p53 wild type (1), Caspex plasmid (deposited by Steven Carr & Samuel Myers (2); Addgene plasmid #97421), dCas9-VP64-p65-Rta (deposited by George Church (3); Addgene plasmid #63798), and Renilla control plasmid pIS1 (deposited by David Bartel; Addgene plasmid #12179). The 2.5 kb FOXQ1 promoter reporter plasmid has been previously described (4). The complete region of FOXQ1 promoter was divided into shorter fragments using the following primers: R1 FW - CATGGGTACC CCACGGCCTGCCATGCCATCT, R1 RV - CATGCTCGAG AAGGCGTTCGTAATGAATGTTTTTCTCT, R2 FW - CATGGGTACC TTATCCCTGGCCAAATCCAAGCC, R2 RV - CATGCTCGAG GTGATCCCCACGCGGCGTCATTAG, R3 FW - CATGGGTACC CTAATGACGCCGCGTGGGGATCAC, R3 RV - CATGCTCGAG TGACCTCTTTCGGGAGCCTTGTGC, R4 FW - CATGGGTACC GCACAAGGCTCCCGAAAGAGGTCA, R4 RV - CATGCTCGAG CCCAGTTCCCCAGCTCCCCAAC, R5 FW - CATGGGTACC GTTGGGGAGCTGGGGAACTG, R5 RV - CATGCTCGAG CGGTTCGACGTTGAGACTTTGGG, R6 FW - CATGGGTACC CCCAAAGTCTCAACGTCGAACCG, R6 RV - CATGCTCGAG AGCCCAGGGAGTCGTCTCC. Missense mutations in the p53 wild-type plasmid and the FOXQ1 promoter reporter were generated by PCR-based mutagenesis (5). All plasmids were validated by partial sequencing (Eurofins Genomics).

RNA interference

Scrambled and small interfering RNAs (siRNAs) were obtained from Integrated DNA Technologies. At least two independent siRNAs for the targets of interest were used. Further validation of gene silencing was performed by immunoblot or qPCR. Cells were transfected with 50 nM siRNAs using Lipofectamine 2000 (Thermo Fisher). Protein or mRNA levels were assessed after 48 hours from transfection.

Western blotting

For immunoblotting, cells were lysed in 0.1% NP-40 in PBS or RIPA buffer with 1x protease inhibitor cocktail. Samples were boiled in Laemmli sample buffer with 50 mM DTT, run on a 10% polyacrylamide gel, transferred to nitrocellulose, and blotted with specific primary antibodies. Primary antibodies were detected using near-infrared (NIR) fluorophore-labelled secondary antibodies (LI-COR) or secondary IgG HRP antibodies (GE Healthcare). Blots were scanned on a LI-COR CLx imager on a ChemiDoc imaging system (BioRad, Hercules, CA) after adding chemiluminescence western blotting reagents (GE Healthcare) when needed. For quantification, proteins of interest were normalised to housekeeping controls reblotted on the same membranes; to illustrate comparable loading, samples were run on separate gels and blotted for housekeeping control only.

Quantitative real-time PCR

RNA isolation was performed using a Qiagen RNeasy mini kit (Hilden, Germany), and reverse transcribed with a Thermo Fisher cDNA synthesis kit. cDNA was amplified using validated custom primers, with SYBR green dye. Data were acquired on a Bio-Rad CFX96 Touch thermocycler (Hercules, USA), and normalised to *HPRT1* or *GAPDH* control.

Genomic locus proteomics and mass spectrometry

Two T175 flasks of 293T-dCas9-APEX2 stable cells were grown for each condition for proteomic experiments. After 5-6 hours from transient transfection of four guide RNAs targeting the human FOXQ1 promoter (sequences and validation data can be found in reference (4)) and the FOXQ1 promoter reporter plasmid, where applicable, cell culture media was replaced by media plus 1 µg/mL doxycycline for 16-18 hours to induce the expression of dCas9-APEX2 protein. Next, cells were treated with media containing 500 µM biotin tyramide phenol (Iris Biotech) in DMSO for 30 min at 37°C, 5% CO_2. Next, 1mM hydrogen peroxide in PBS was added to induce biotinylation. After 60 seconds of very gentle swirling, the solution was discarded, and the cells were washed three times with ice cold PBS containing 100 mM sodium azide, 100 mM sodium ascorbate and 50 mM TROLOX (6-hydroxy-2,5,7,8-tetramethylchroman-2-carboxylic acid). Cells from two T175 flasks were scraped and pooled in 50 ml Falcon tubes and washed three additional times with ice cold PBS to remove the excess of biotin. Nuclear protein extraction was performed using 0.1% NP-40 in PBS with 1x protease inhibitor cocktail.

Biotinylated proteins were precipitated overnight with streptavidin beads (GE Healthcare) at 4°C with end-over-end rotation. The beads were washed four times with 50 mM ammonium bicarbonate (NH_4HCO_3) and digested using spectrometry-grade trypsin (Thermo Fisher) at 37°C with end-over-end rotation. The digested samples were dried by vacuum centrifugation. Samples were analyzed by mass spectrometry using an Easy nano LC 1200 system interfaced with a nanoEasy spray ion source (Thermo Fisher Scientific) connected Q Exactive HF Hybrid Quadrupole-Orbitrap Mass Spectrometer (Thermo Fisher Scientific).

Raw data were processed by Proteome Discover 2.5 (Thermo Fisher Scientific) searching against the *Homo sapiens* UniProt database (release from 2019-12-16) with Sequest HT search engine. The search parameters were: Taxonomy: *Homo sapiens*; Enzymes; trypsin with two missed cleavages, no variable or fixed Modifications; Peptide Mass Tolerance, 10 ppm; MS/MS Fragment Tolerance, 0.02 Da. Quantification of the analysed data was performed with Scaffold 5.0.0 (Proteome Software), a Proteome Software using total spectral count. Protein identifications that were accepted contained at least one identified peptides and probability >95%. Peptide identifications were accepted if they could be established at >90% probability by the Scaffold Local FDR algorithm.

Chromatin immunoprecipitation

HCT116 cells (5×10^7) were crosslinked in 1% formaldehyde in PBS for 20 min, to preserve DNA-protein interactions. The reaction was blocked with 125 mM glycine (final conc.) and the cells were lysed in 1 ml HEPES lysis buffer (0.3% SDS, 1% Triton-X 100, 0.15 M NaCl, 1 mM EDTA, 0.5 mM EGTA, 20 mM HEPES) with 1x protease inhibitor. Samples were sonicated in a QSonica Q700 Sonicator with a cup horn immersed in ice-cold water. The sonication program used was: 50 cycles, 30 sec ON/ 1 min OFF at amplitude 90. The sonicated chromatin was diluted twice to 0.15% SDS and cell debris were discarded by centrifugation at 14,000 g for 10 min. The supernatant was transferred to a new tube and 1% input was collected. The chromatin was incubated overnight with end-over-end rotation at 4°C with 50 µl Protein A/G agarose beads plus 5 µg of mouse IgG or mouse anti-P53 antibody. Next, the beads were washed at 4°C with wash buffer 1 (0.1% SDS, 0.1% deoxycholate, 1% Triton X-100, 0.15 M NaCl, 1 mM EDTA, 0.5 mM EGTA, 20 mM HEPES), wash buffer 2 (0.1% SDS, 0.1% sodium deoxycholate, 1% Triton X-100, 0.5 M NaCl, 1 mM EDTA, 0.5 mM EGTA, 20 mM HEPES), wash buffer 3 (0.25 M LiCl, 0.5% sodium deoxycholate, 0.5% NP-40, 1 mM EDTA, 0.5 mM EGTA, 20 mM HEPES) and twice with Tris EDTA buffer (all washing solution were kept at 4°C). The chromatin was eluted with 1% SDS, 0.1 M NaHCO3 and de-crosslinked by incubation at 65°C overnight with 200 mM NaCl. Then, DNA was extracted with phenol-chloroform and ethanol precipitated. The immunoprecipitated DNA was used for quantitative PCR using the following primers: R3 FW – GGAGCCCATTTAGTGAGCAA, R3 RV – GCTCCTTTCAAGGACCATCTT,

R5 FW - ACCCCTCCTGGGCTCTTTA, R5 RV – CGGTTCGACGTTGAGACTTT, NC FW – CTACTCGTACATCGCGCTCA, NC RV – GCGGAAAAAGGGGAACTTG.

Luciferase assays

Cells were plated onto 96-well plates. The next day, cells were transiently transfected with firefly luciferase reporter constructs and Renilla luciferase control plasmids using jetOPTIMUS transfection reagents (Polyplus Transfection, Illkirch, France), according to the supplier's recommendations. Cells were lysed in passive lysis buffer (25 mM Tris, 2 mM DTT, 2 mM EDTA, 10% (v/v) glycerol, 1% (v/v) Triton X-100, (pH 7.8)) and agitated on a plate shacker for 10 min. Firefly luciferase buffer (200 µM D-luciferin in 200 mM Tris-HCl, 15 mM MgSO4, 100 µM EDTA, 1 mM ATP, 25 mM DTT, pH 8.0) was added to each well and the plate was incubated for 2 min at room temperature. Luciferase activity was measured using a SpectraMax iD3 Multi-Mode Microplate Reader (Molecular Devices). Next, Renilla luciferase buffer (4 µM coelenterazine-h in 500 mM NaCl, 500 mM Na2SO4, 10 mM NaOAc, 15 mM EDTA, 25 mM sodium pyrophosphate, 50 µM phenyl-benzothiazole, pH 5.0) was added to the plate and luminescence was measured immediately. Data were normalized to the Renilla control values, performed in triplicate.

Quantitative real-time PCR

RNA isolation was performed using a Qiagen RNeasy mini kit (Hilden, Germany), and reverse transcribed with a Thermo Fisher cDNA synthesis kit. cDNA was amplified using validated custom primers, with SYBR green dye. Data were acquired on a Bio-Rad CFX96 Touch thermocycler (Hercules, USA), and normalised to *HPRT1* or *GAPDH* control.

CRISPR activation assay

CRISPR/Cas9-mediated activation of *FOXQ1* was performed essentially as described in reference (4). Briefly, HCT116 were transiently transfected with dCas9-VP64-p65-Rta and pooled FOXQ1 gRNAs described under "Genomic locus proteomics and mass spectrometry". After 48 hours, cells were lysed for mRNA extraction.

In silico p53 binding prediction

Position weight matrices for human p53 (MA0106.1, MA0106.2, MA0106.3) were obtained from JASPAR 2022 (6), and aligned to the FOXQ1 promoter sequence (-607 to +173) using the R package TFBSTools v1.38.0 (7). Search parameters were: minimum score 75%, empirical p-value < 0.001 (using the TFMPvalue method implemented in TFBSTools).

Public data analysis

The following public datasets were used for analysis: Cancer Cell Line Encyclopedia (Broad Institute (8); 1739 samples, 60% *TP53* mutant), Pan-cancer analysis of whole genomes (ICGC/TCGA (9, 10); 2922 samples, 33% *TP53* mutant), Colorectal Adenocarcinoma (TCGA (9, 10), a subset of the Pan-cancer analysis of whole genomes; 594 samples, 53% *TP53* mutant). All data were accessed and pre-processed in the cBioPortal for Cancer Genomics (11). Additional analyses and visualisation were performed in R v4.2.1. Overlapping samples and patients within each individual dataset were removed before analysis. Any sample with annotated *TP53* or *APC* genetic alteration was considered to be mutated for this gene irrespective of the nature of the mutation. In the Colorectal Adenocarcinoma dataset, we additionally removed samples with other common activating Wnt pathway mutations (*AXIN1*, *TCF7L2*, *CTNNB1*, *RNF43*, *ZNRF3*) and missing gene expression data, resulting in 439 samples. Inter-group differences were analysed using the TukeyHSD function in R. Survival data were processed and visualised using the R packages survival v3.5 and survminer v0.4.9, with data stratified by the median *FOXQ1* expression (batch-normalised RSEM) across all samples. Statistical significance was determined using the log-rank test. Public p53 ChIP-seq data were retrieved from the ReMap 2022 database (12), and mapped to the human FOXQ1 promoter using ReMapEnrich v0.99.0 in R.

Statistical analysis

Data are shown as mean with standard deviation. Each experiment included controls (e.g., empty backbone plasmid, scrambled siRNA and substance carriers) at identical concentrations. Statistical tests are indicated in the figure legends and were carried out in R v4.2.1.

REFERENCES

1. Xirodimas, D., Saville, M., Edling, C., Lane, D., andLáin, S. (2001) Different effects of p14ARF on the levels of ubiquitinated p53 and Mdm2 in vivo Oncogene **20**, 4972-4983,
2. Myers, S. A., Wright, J., Peckner, R., Kalish, B. T., Zhang, F., andCarr, S. A. (2018) Discovery of proteins associated with a predefined genomic locus via dCas9-APEX-mediated proximity labeling Nat Methods **15**, 437-439 10.1038/s41592-018-0007-1
3. Chavez, A., Scheiman, J., Vora, S., Pruitt, B. W., Tuttle, M., E, P. R. I. *et al.* (2015) Highly efficient Cas9-mediated transcriptional programming Nat Methods **12**, 326-328 10.1038/nmeth.3312
4. Pizzolato, G., Moparthi, L., Soderholm, S., Cantu, C., andKoch, S. (2022) The oncogenic transcription factor FOXQ1 is a differential regulator of Wnt target genes J Cell Sci **135**, 10.1242/jcs.260082
5. Edelheit, O., Hanukoglu, A., andHanukoglu, I. (2009) Simple and efficient site-directed mutagenesis using two single-primer reactions in parallel to generate mutants for protein structure-function studies BMC biotechnology **9**, 1-8,

6. Castro-Mondragon, J. A., Riudavets-Puig, R., Rauluseviciute, I., Berhanu Lemma, R., Turchi, L., Blanc-Mathieu, R. *et al.* (2021) JASPAR 2022: the 9th release of the open-access database of transcription factor binding profiles Nucleic Acids Research **50**, D165-D173 10.1093/nar/gkab1113
7. Tan, G., andLenhard, B. (2016) TFBSTools: an R/bioconductor package for transcription factor binding site analysis Bioinformatics **32**, 1555-1556 10.1093/bioinformatics/btw024
8. Ghandi, M., Huang, F. W., Jané-Valbuena, J., Kryukov, G. V., Lo, C. C., McDonald, E. R., 3rd *et al.* (2019) Next-generation characterization of the Cancer Cell Line Encyclopedia Nature **569**, 503-508 10.1038/s41586-019-1186-3
9. ICGC/TCGA Pan-Cancer Analysis of Whole Genomes Consortium (2020) Pan-cancer analysis of whole genomes Nature **578**, 82-93 10.1038/s41586-020-1969-6
10. Hoadley, K. A., Yau, C., Hinoue, T., Wolf, D. M., Lazar, A. J., Drill, E. *et al.* (2018) Cell-of-Origin Patterns Dominate the Molecular Classification of 10,000 Tumors from 33 Types of Cancer Cell **173**, 291-304.e296 10.1016/j.cell.2018.03.022
11. Gao, J., Aksoy, B. A., Dogrusoz, U., Dresdner, G., Gross, B., Sumer, S. O. *et al.* (2013) Integrative analysis of complex cancer genomics and clinical profiles using the cBioPortal Science signaling **6**, pl1-pl1,
12. Hammal, F., de Langen, P., Bergon, A., Lopez, F., andBallester, B. (2021) ReMap 2022: a database of Human, Mouse, Drosophila and Arabidopsis regulatory regions from an integrative analysis of DNA-binding sequencing experiments Nucleic Acids Research **50**, D316-D325 10.1093/nar/gkab996

SUPPLEMENTAL FIGURES

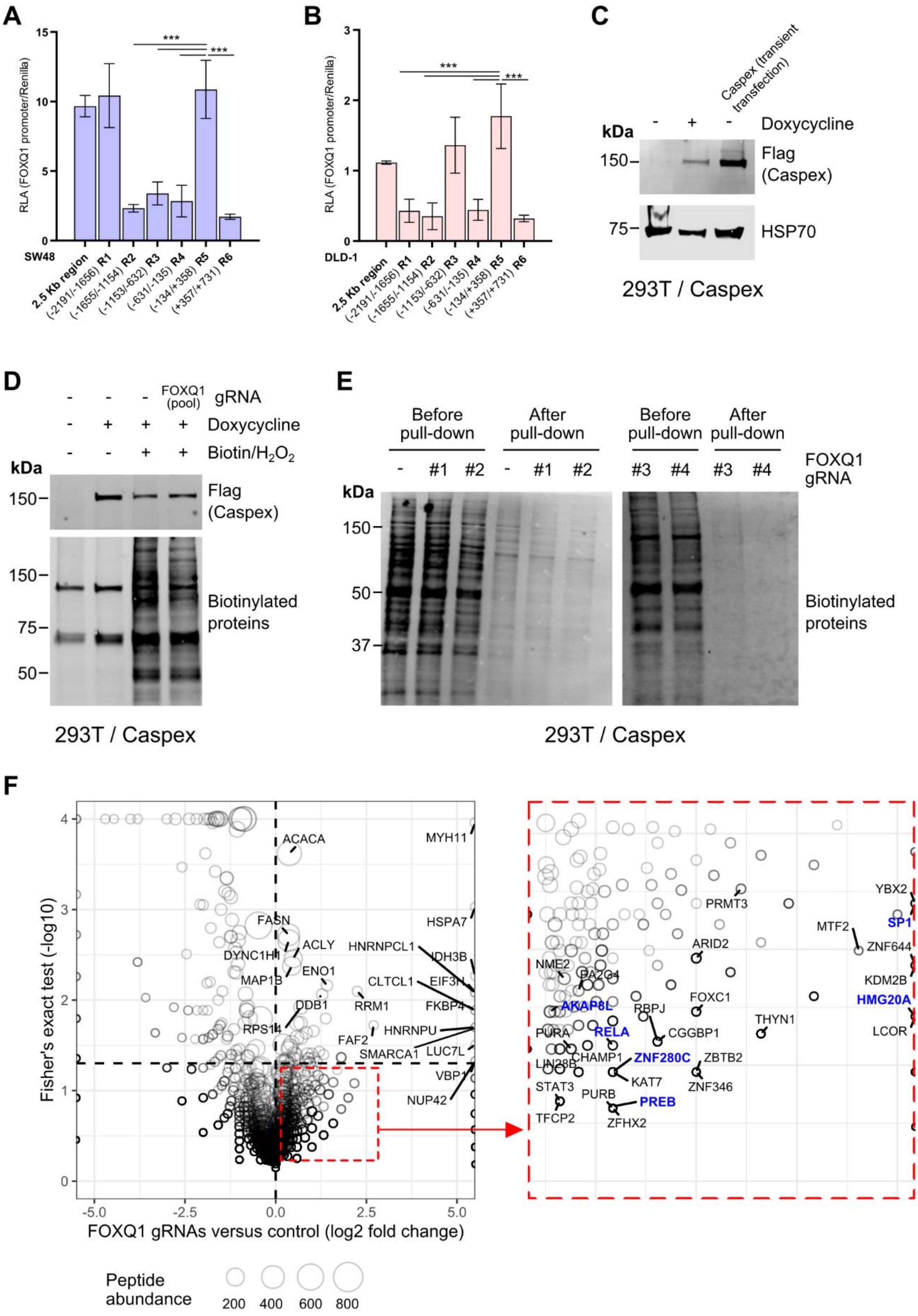

Fig. S1: Additional controls for FOXQ1 promoter reporter and GLoPro experiments. S1A, B FOXQ1 promoter activity assay in SW48 and DLD-1 cells. Mean and SD of normalized luciferase values of n=2 independent experiment (ANOVA with Tukey's post-hoc test, ***p < 0.001). **S1C** Immunoblot analysis of the induction of Flag-tagged Caspex plasmid in the 293T stable cell line after treatment with 500 ng/mL doxycycline for ~18 hours. Transient overexpression using the Caspex plasmid was used as control. **S1D** Immunoblot analysis to validate the Caspex-induced protein biotinylation. Detection of Caspex with anti-Flag antibodies was performed after treatment with 500 ng/mL doxycycline for ~18 hours. Next, cells were incubated with 500 µM biotin tyramide for 30 min and treated with 1 mM hydrogen peroxide for 1 min to induce biotinylation. **S1E** Immunoblot analysis to control for protein biotinylation in the presence of Caspex guided to the FOXQ1 promoter by four gRNAs versus the no guide control. Lysates were run before and after pull-down with streptavidin beads. **S1F** Volcano plot of GLoPro results in untransfected 293T / Caspex cells. The magnified box on the right highlights transcription factors enriched at the endogenous FOXQ1 promoter. Genes in blue were validated by RNA interference.

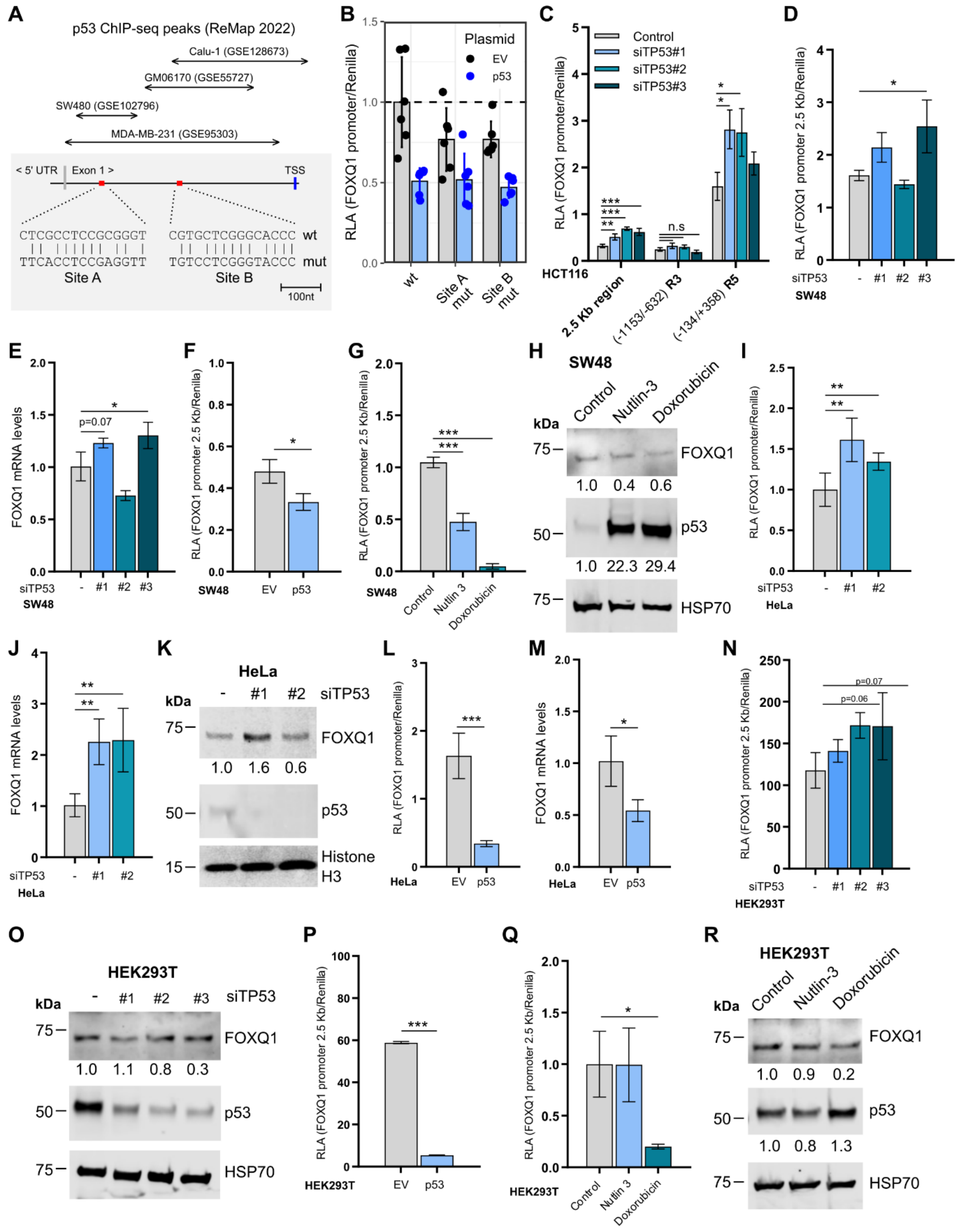

Fig. S2: p53 is a negative regulator of *FOXQ1* expression. S2A Schematic representation of the human FOXQ1 promoter covering the R5 region. Red boxes indicate putative p53 binding sites identified in silico. Mutations (mut) introduced in the 2.5kb promoter reporter plasmid are shown below. Double-headed arrows above the schematic highlight p53 ChIP-seq hits curated in the ReMap 2022 database. Genomic ranges are drawn to scale. TSS, transcription start site. **S2B** FOXQ1 promoter reporter assay

using the wild-type and mutated 2.5kb reporter plasmid. **S2C** Luciferase assay in HCT116 cells using the FOXQ1 promoter reporters upon silencing of *TP53* with three independent siRNAs. n=2 independent experiment (ANOVA with Dunnett´s post-hoc test, ***p < 0.001 **p < 0.01 *p < 0.05 or n.s = not significant). **S2D** Luciferase assay in SW48 cells using the FOXQ1 reporter plasmid upon silencing of *TP53*. n=3 independent experiment (ANOVA with Dunnett´s post-hoc test, *p < 0.05). **S2E** qPCR analysis of *FOXQ1* mRNA levels in SW48 cells upon silencing of *TP53*. FOXQ1 gene expression was normalized to *HPRT1* housekeeping gene (ANOVA with Dunnett´s post-hoc test, *p < 0.05). **S2F** Luciferase assay in SW48 cells using the FOXQ1 reporter plasmid after overexpression of p53 wild-type plasmid. The graph shows mean and SD of normalized luciferase values of n=2 independent experiments (Welch's t-test, p < 0.05). **S2G** Luciferase assay in SW48 cells using the FOXQ1 reporter plasmid after treatment with 10 µM Nutlin-3 or 1 µM doxorubicin for 24 hours. Mean and SD of normalized luciferase values of n=3 independent experiments (ANOVA with Dunnett´s post-hoc test, ***p < 0.001). **S2H** Immunoblot analysis of FOXQ1 protein levels after treatment with 10 µM Nutlin-3 or 1 µM doxorubicin for 24 hours. Normalised relative band intensities are indicated. **S2I** Luciferase assay in HeLa cells using the FOXQ1 reporter plasmid upon silencing of *TP53*. n=3 independent experiment (ANOVA with Dunnett´s post-hoc test, **p < 0.01). **S2J** qPCR analysis of *FOXQ1* mRNA levels in HeLa cells upon silencing of *TP53*. FOXQ1 gene expression was normalized to *GAPDH* housekeeping gene (ANOVA with Dunnett´s post-hoc test, **p < 0.01). **S2K** Immunoblot analysis of FOXQ1 protein levels in HeLa cells upon silencing of *TP53*. FOXQ1 levels were normalized to Histone H3 loading control. **S2L** Luciferase assay in HeLa cells using the FOXQ1 reporter plasmid upon overexpression of p53 wild-type plasmid. n=3 independent experiment (Welch's t-test, ***p < 0.001). **S2M** qPCR analysis of *FOXQ1* mRNA levels in HeLa cells upon overexpression of p53 wild-type plasmid. FOXQ1 gene expression was normalized to *GAPDH* housekeeping gene (Welch's t-test, *p < 0.05). **S2N** Luciferase assay in 293T cells using the full-length FOXQ1 reporter plasmid upon silencing of *TP53* (ANOVA with Dunnett´s post-hoc test). **S2O** Immunoblot analysis of FOXQ1 protein levels in 293T upon silencing of *TP53*. **S2P** Luciferase assay in 293T cells using the FOXQ1 reporter plasmid after overexpression of p53 wild-type plasmid. Mean and SD of normalized luciferase values of n=2 independent experiments (Welch's t-test, ***p < 0.001). **S2Q** Luciferase assay in 293T cells using the FOXQ1 reporter plasmid after treatment with 10 µM Nutlin-3 or 1 µM doxorubicin for 24 hours. Mean and SD of normalized luciferase values of n=2 independent experiments (ANOVA with Dunnett´s post-hoc test, *p < 0.05). **S2R** Immunoblot analysis of FOXQ1 protein in 293T upon treatment with Nutlin-3 and doxorubicin.

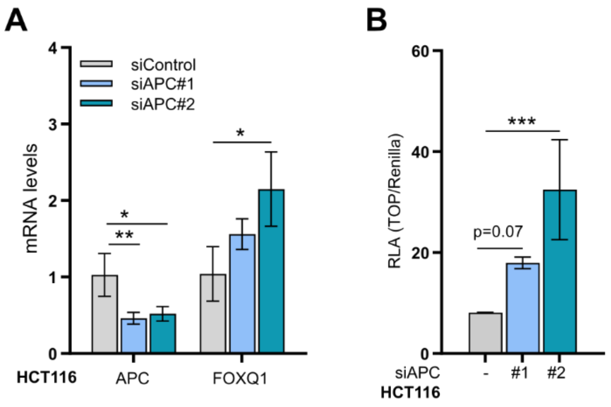

Fig. S3: FOXQ1 is a target of the Wnt/β-catenin pathway. S3A qPCR analysis of *APC* and *FOXQ1* mRNA levels in HCT116 cells (ANOVA with Dunnett´s post-hoc test, **p < 0.01 *p < 0.05). **S3B** β-catenin/TCF reporter (TOPflash) assay upon silencing of *APC* by 2 independent siRNAs (ANOVA with Dunnett´s post-hoc test, ***p < 0.001).
